# Functional lacrimal gland regeneration by transplantation of a bioengineered organ germ

**DOI:** 10.1038/ncomms3497

**Published:** 2013-10-01

**Authors:** Masatoshi Hirayama, Miho Ogawa, Masamitsu Oshima, Yurie Sekine, Kentaro Ishida, Kentaro Yamashita, Kazutaka Ikeda, Shigeto Shimmura, Tetsuya Kawakita, Kazuo Tsubota, Takashi Tsuji

**Affiliations:** 1 Department of Ophthalmology, School of Medicine, Keio University, Shinjuku-ku, Tokyo 160 8582, Japan; 2 Research Institute for Science and Technology, Tokyo University of Science, Noda, Chiba 278 8510, Japan; 3 Division of Research and Development, Organ Technologies Inc., Chiyoda-ku, Tokyo 101 0048, Japan; 4 Department of Biological Science and Technology, Graduate School of Industrial Science and Technology, Tokyo University of Science, Noda, Chiba 278 8510, Japan; 5 Institute for Advanced Biosciences, Keio University, Tsuruoka, Yamagata 997 0035, Japan

## Abstract

The lacrimal gland has a multifaceted role in maintaining a homeostatic microenvironment for a healthy ocular surface via tear secretion. Dry-eye disease, which is caused by lacrimal gland dysfunction, is one of the most prevalent eye diseases that cause corneal epithelial damage and results in significant loss of vision and a reduction in the quality of life. Here we demonstrate orthotopic transplantation of bioengineered lacrimal gland germs into adult mice with an extra-orbital lacrimal gland defect, a mouse model that mimics the corneal epithelial damage caused by lacrimal gland dysfunction. The bioengineered lacrimal gland germs and harderian gland germs both develop *in vivo* and achieve sufficient physiological functionality, including tear production in response to nervous stimulation and ocular surface protection. This study demonstrates the potential for bioengineered organ replacement to functionally restore the lacrimal gland.

## Abstract (web summary)

Lacrimal glands maintain a healthy corneal epithelium but are dysfunctional for example in dry-eye disease. Here, the authors transplant bioengineered lacrimal and harderian gland germs into mice, where they connect to the host duct and nervous system and restore lacrimal gland function.

## Graphical abstract

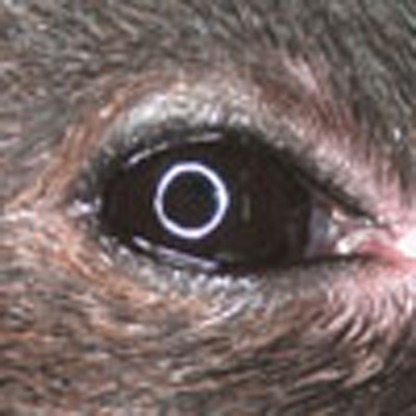

Lacrimal glands, which consist of a main gland and small accessory glands, have a multifaceted role in maintaining a homeostatic microenvironment for a healthy ocular surface through tear secretion^1^. The main lacrimal gland, which is organized according to a tubuloalveolar scheme that includes acini, ducts and myoepithelial cells, develops from its organ germ by induction from reciprocal epithelial and mesenchymal interactions during embryogenesis^2^. The tear film is a trilaminar fluid composed of a superficial lipid layer, an intermediate aqueous layer and an underlying mucous layer. This film covers the entire ocular surface, including the bulbar and palpebral conjunctiva, and the cornea^3^. The aqueous tear layer is produced by the lacrimal glands and contains water and various tear proteins, such as lactoferrin and lipocalin, which have several functions, including moisturising and antimicrobial activity^3^,^4^. The lipid layer, which has an important role in retarding water evaporation, is secreted by the meibomian gland in humans and the harderian gland in murine species^5^,^6^,^7^. Tears are indispensable to lid lubrication, protection of the epithelial surface and visual function.

Dry-eye disease (DED), which is caused by tear shortage, results from lacrimal gland dysfunction caused by systemic diseases and environmental exposures, such as Sjogren’s syndrome and ocular cicatricial pemphigoid, or from other causes, including aging, long-term work with a visual display, dry room environments, the use of contact lenses and refractive surgery^8^,^9^,^10^. DED is one of the most prevalent eye diseases leading to corneal epithelial damage, which is characterized by the loss of individual cells from the superficial cell layer of the corneal epithelium. DED is diagnosed by a punctate pattern of fluorescence staining at the ocular surface^11^. The irregularity of the ocular surface, which is caused by the corneal epithelial damage, results in ocular discomfort, significant loss of vision and a decrease in the quality of life^12^. Current therapies for DED, including artificial tear solutions, are transient and do not completely substitute for the normal tear complex, which is composed of water, salts, hydrocarbons, proteins and lipids^13^,^14^,^15^. Several therapeutic approaches have been developed to restore lacrimal gland function, including heterotopic salivary gland transplantation^16^ and regenerative medicine^17^.

The current state of the art techniques in regenerative therapy use stem cell transplantation therapy to repair damaged tissue^18^,^19^. These methods use tissue-derived stem cells to treat various diseases, including hematopoietic malignancies^20^, myocardial infarction^21^ and hepatic insufficiency^22^. Candidates for secretory organ-derived stem cells are intercalated duct cells, human c-kit-positive duct cells and salivary gland-derived progenitor cells in duct-ligated or irradiated salivary gland model animals^23^,^24^,^25^. Stem cell transplantation therapy using lacrimal gland-derived progenitor cells, which are involved in the repair of impaired lacrimal glands, has been investigated for the recovery of lacrimal gland impairment^17^,^26^. In addition, a novel concept for organ-replacement regenerative therapy has been recently proposed to replace organs lost or damaged by disease, injury or aging^27^,^28^,^29^. We have demonstrated the successful replacement of entire and fully functioning bioengineered ectodermal organs, such as teeth and hair follicles, through coordination with peripheral tissues, such as nerves, by orthotopic engraftment of bioengineered organ germ.

Here we report functional bioengineered lacrimal gland replacement via an orthotopic engraftment of a bioengineered lacrimal gland germ into an extra-orbital lacrimal gland-defect mouse model, a model that mimics ocular-surface damage by lacrimal gland dysfunction. The bioengineered lacrimal and harderian gland germs, both developed *in vivo*, achieved sufficient physiological functions, including tear production in response to pilocarpine and menthol stimulation and ocular-surface protection. Our current study thus provides the potential for bioengineered lacrimal gland replacement to restore lacrimal gland function.

## Results

### Generating bioengineered lacrimal and harderian gland germs

**Figure 1:**
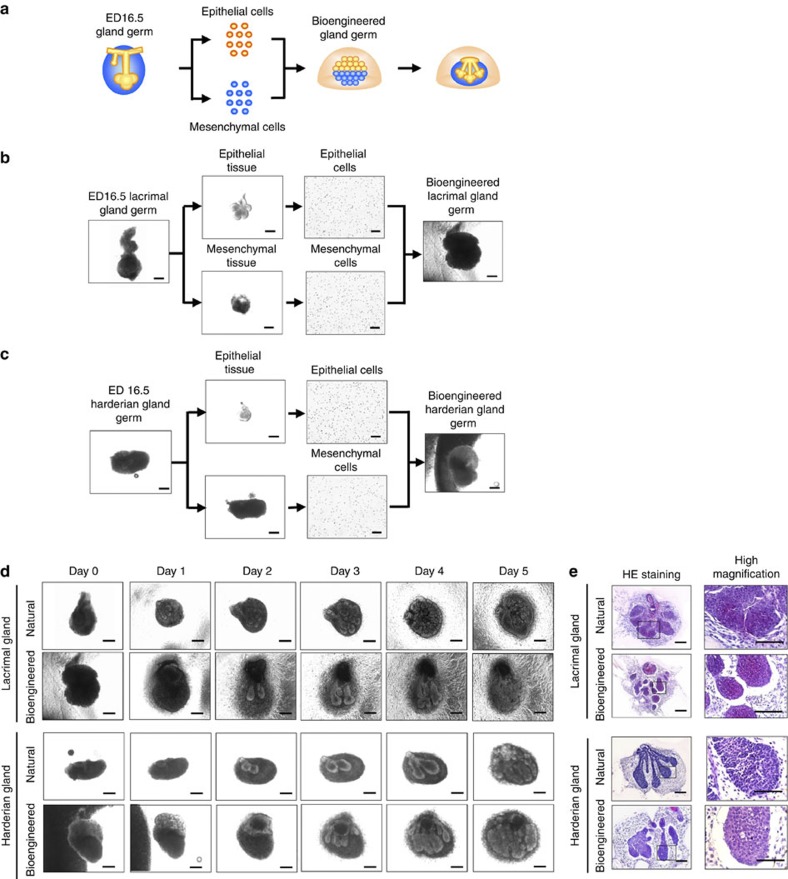
Development and engraftment of the bioengineered gland. (**a**) Schematic representation of the methods used to generate a bioengineered lacrimal or harderian gland germ. (**b**) Phase-contrast images of the regeneration of the bioengineered lacrimal gland germ by the organ germ method. Scale bar, 100 μm. (**c**) Phase-contrast images of the development of harderian gland germ in organ culture. Scale bar, 100 μm. (**d**) Phase-contrast images of the development of natural and bioengineered lacrimal gland germ (upper) and harderian gland germ (lower) in organ culture. Scale bar, 100 μm. (**e**) Light microscopic images of HE-stained organ cultures of a natural and a bioengineered lacrimal gland germ (upper) and a harderian gland germ (lower) on day 3 and corresponding higher magnification images (right). Scale bar, 100 μm.

We first investigated whether bioengineered lacrimal gland and harderian gland germs could be reconstituted using our previously developed organ-germ method (Fig. 1a)^27^. We used dissociated single cells from the epithelial and mesenchymal tissues of embryonic day-16.5 murine-lacrimal gland germs and harderian gland germs to reconstitute bioengineered lacrimal gland and harderian gland germs (Fig. 1b,c). After 1 day in organ culture, epithelial–mesenchymal interactions appeared to have occurred in both of the bioengineered gland germs, which had developed to the initial bud stage. After 3 days in organ culture, the bioengineered lacrimal gland germ and the harderian gland germ had both undergone branching morphogenesis, followed by stalk elongation and cleft formation (Fig. 1d,e). These results indicated that bioengineered lacrimal glands and harderian glands could be reconstituted using the organ-germ method.

### Engraftment and development of a bioengineered lacrimal gland

**Figure 2:**
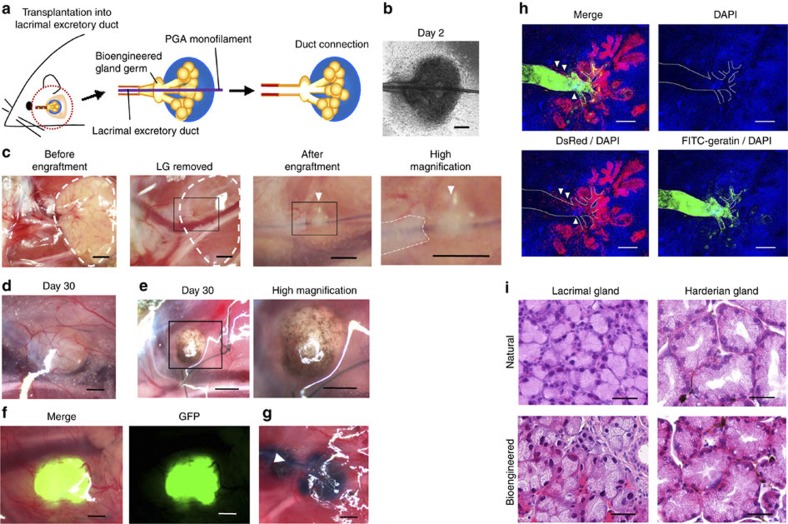
Engraftment of the bioengineered gland germ. (**a**) Schematic representation of the methods used to engraft a bioengineered germ into an adult lacrimal gland-defect mouse. (**b**) Phase-contrast images of bioengineered lacrimal gland germs with a PGA monofilament inserted after 2 days of culture. Scale bar, 100 μm. (**c**) Photographs of procedures for bioengineered gland germ engraftment. The extra-orbital lacrimal gland was completely removed (left, centre-left, white dotted line). The boxed area in the centre-left panel is the engraftment site shown at a higher magnification in the centre-right panel. The boxed area in the centre-right panel is shown at a higher magnification in the right panel. The PGA monofilament was inserted into the host lacrimal excretory duct (centre-right, right). The white dotted line in the right panel indicates the host lacrimal duct. The arrowhead in the centre-right and right panels indicates the engrafted bioengineered lacrimal gland with a PGA monofilament. Scale bar, 1 mm. (**d**) Photographs of bioengineered lacrimal glands at 30 days after engraftment (left). Scale bar, 500 μm. (**e**) Photographs of a bioengineered harderian gland at 30 days after engraftment (left). The boxed area in the left panel is shown at a higher magnification in the right panel. Scale bar, 500 μm. (**f**) Photographs of a bioengineered lacrimal gland reconstituted using epithelial cells and mesenchymal cells from GFP-transgenic mice. A merged image (left) and a fluorescent image (right) are shown. Scale bar, 500 μm. (**g**) Analysis of the duct connection using Evans blue dye injection. The arrowhead indicates the injection site. Scale bar, 500 μm. (**h**) Histological analysis of the duct connection. 3D images of the bioengineered lacrimal gland reconstituted between DsRed transgenic mice-derived epithelial cells (red) and normal mice-derived mesenchymal cells. Bioengineered lacrimal glands developed *in vivo* with the correct connection (arrowhead) to the recipient lacrimal excretory duct. FITC-gelatin conjugate (green), DAPI (blue) and excretory duct (dotted line) are shown. Scale bar, 100 μm. (**i**) HE-stained lacrimal gland (left) and harderian gland (right). A natural (upper) and bioengineered (lower) gland after 30 days of engraftment are shown. Scale bar, 50 μm.

To induce ductal associations between the epithelium of the host excretory lacrimal duct and the bioengineered lacrimal or harderian gland germs, we used our previously developed interepithelial, tissue-connecting plastic device, which employs a polyglycolic acid (PGA) monofilament inserted into the bioengineered germ to direct the infundibulum (Fig. 2a,b). The bioengineered lacrimal and harderian gland germs were then engrafted in the correct orientation into the lacrimal duct of a 7-week-old (adult) extra-orbital lacrimal gland-defect model mouse (Fig. 2c). Within 30 days after engraftment of the bioengineered germs, transplant growth was apparent (Fig. 2d). Macroscopic observation revealed that the engrafted bioengineered harderian gland had a brown-pigmented surface, which is a characteristic feature of the harderian glands (Fig. 2e)^6^. The bioengineered lacrimal and harderian glands could develop *in vitro* with the frequencies of 95.0% and 93.8%, respectively. The success rates for the development of engrafted bioengineered lacrimal and harderian glands were 77.8% and 73.7%, respectively. We also engrafted non-transgenic mice with green-fluorescence protein (GFP)-labelled bioengineered lacrimal gland germs that were reconstituted from lacrimal gland germ-derived epithelial and mesenchymal cells from GFP-transgenic mice. The GFP-labelled, bioengineered lacrimal glands were regenerated and could be observed in the engrafted area of the adult mice (Fig. 2f). Excretory ducts that send secreted fluid to the target area are essential elements of the secretory glands. We next investigated whether the engrafted bioengineered lacrimal gland could connect with the lacrimal duct in the adult lacrimal gland-defect model mice. We confirmed that Evans blue dye injected into the host lacrimal duct reached the engrafted bioengineered lacrimal gland without leaking (Fig. 2g). We also engrafted Discosoma sp. (DsRed)-labelled bioengineered lacrimal gland germs, which were reconstituted between DsRed transgenic mouse-derived epithelial cells and normal mouse-derived mesenchymal cells, for 14 days and then injected the Fluorescein 5-isothiocynate (FITC)–gelatin conjugate into the host lacrimal excretory duct. The duct connection between the DsRed-labelled bioengineered lacrimal gland and the recipient’s excretory duct was histologically observed (Fig. 2h). These findings indicated that the bioengineered lacrimal gland had successfully connected to the excretory duct of the recipient mouse. We next analysed the histological structures of the engrafted bioengineered glands. Lobules consisting of acinar cells and ducts were observed in the bioengineered lacrimal gland using HE staining and were similar to those of normal glands (Fig. 2i). The bioengineered harderian glands had acini with large lumens and displayed brown pigment in the interstitial tissue, both of which are characteristic features of the natural harderian gland structure^6^ (Fig. 2i). The average±s.e.m of the maximum acini diameter were 48.6±1.5 and 70.9±1.7 μm in the bioengineered lacrimal and harderian glands, respectively, and 46.0±1.2 and 71.3±1.6 μm in the natural lacrimal and harderian glands, respectively. These results indicated that the engrafted bioengineered gland germ was accepted by the host and developed at the engraftment site, achieving a connection with the recipient duct.

### Histology of bioengineered lacrimal and harderian glands

**Figure 3:**
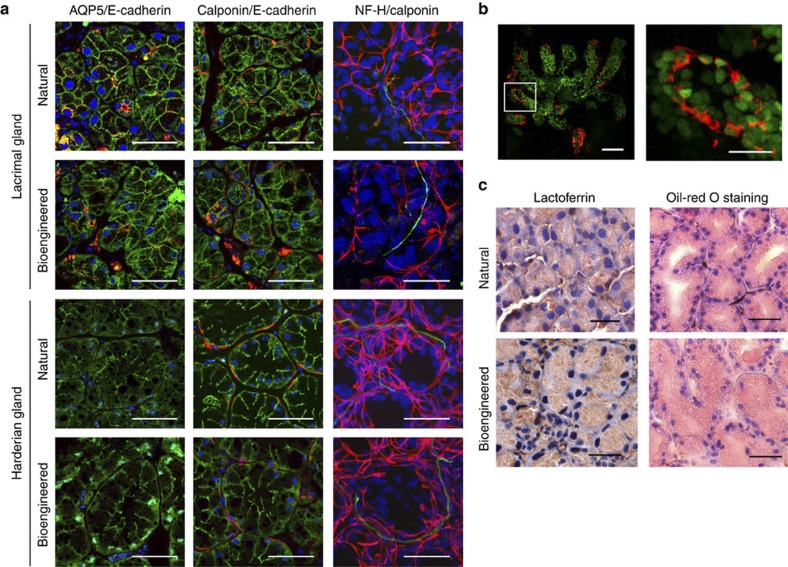
Histological analysis of a bioengineered gland. (**a**) Immunohistochemical analysis of the acinar and duct cells in natural or bioengineered lacrimal and harderian glands. Their respective acini were analysed by immunostaining with specific antibodies for AQP5(red, in left panels), calponin (red, in centre and left panels), neurofilament-H (NF-H, green, in right panels) and E-cadherin (green, in left and centre panels). Nuclei were stained using Hoechst 33342 dye (blue). Scale bar, 50 μm. (**b**) Immunohistochemical analysis of a bioengineered lacrimal gland reconstituted between H2B-GFP-transgenic mice-derived epithelial cells and normal mice-derived mesenchymal cells. Green fluorescence indicates the nuclei of epithelial cells isolated from H2B-GFP-transgenic mice. The myoepithelial cells were analysed by immunostaining with specific antibodies for calponin (red). The boxed area in the left panel is shown at a higher magnification in the right panel. Scale bar, 50 μm in the upper panel, 20 μm in the lower panel. (**c**) Photomicrographs showing lactoferrin (left) in the acini of a natural lacrimal gland (upper) and a bioengineered lacrimal gland (lower). Lipids (right) in the natural harderian gland (upper) and bioengineered harderian gland (lower) acini were revealed by oil-red O staining. Scale bar, 50 μm.

We next analysed the three-dimensional (3D) structures and coordination of the transplanted bioengineered glands. Histological analysis revealed that acinar and duct cells in the bioengineered lacrimal and harderian glands were found to express E-cadherin (Fig. 3a, left). Aquapolin-5 (AQP5), a membrane water-channel protein expressed on the ductal and apical membranes of acini that has an essential role in fluid secretion, was present in both bioengineered and natural lacrimal glands (Fig. 3a, left). Acinar and duct cells in the bioengineered lacrimal and harderian glands contained calponin-expressing myoepithelial cells that enveloped the acini, similar to the structure observed in natural glands (Fig. 3a, centre). Antineurofilament-immunoreactive nerve fibres were detected in the interstitial tissue among the acini of the bioengineered lacrimal glands and the fibres connected to the calponin-positive myoepithelial cells, as seen in the natural glands (Fig. 3a, right). These results indicated that the transplanted bioengineered gland germ achieved the correct 3D structure and received nerve invasion following transplantation. Furthermore, analyses of the bioengineered lacrimal gland reconstituted between H2B-GFP-transgenic mice-derived epithelial cells and normal mice-derived mesenchymal cells revealed that the acinar and duct cells were derived from epithelial cells (Fig. 3b). The myoepithelial cells are also derived from the epithelial cells because the cells have the FITC-labelled nuclei and calponin protein (Fig. 3b). We next investigated whether the bioengineered lacrimal and harderian gland acini could produce the characteristic secretary substances. The expression of lactoferrin, a protein secreted by the lacrimal gland, was found in the acini of bioengineered lacrimal glands (Fig. 3c). We used oil-red O staining to confirm that lipids, which are secreted mainly by the harderian gland, were present in the acini of the bioengineered harderian glands (Fig. 3c). These results indicated that the bioengineered lacrimal and harderian glands would likely secrete the appropriate tear contents in response to neural stimulus.

### Secretion of bioengineered tear and lipids

**Figure 4:**
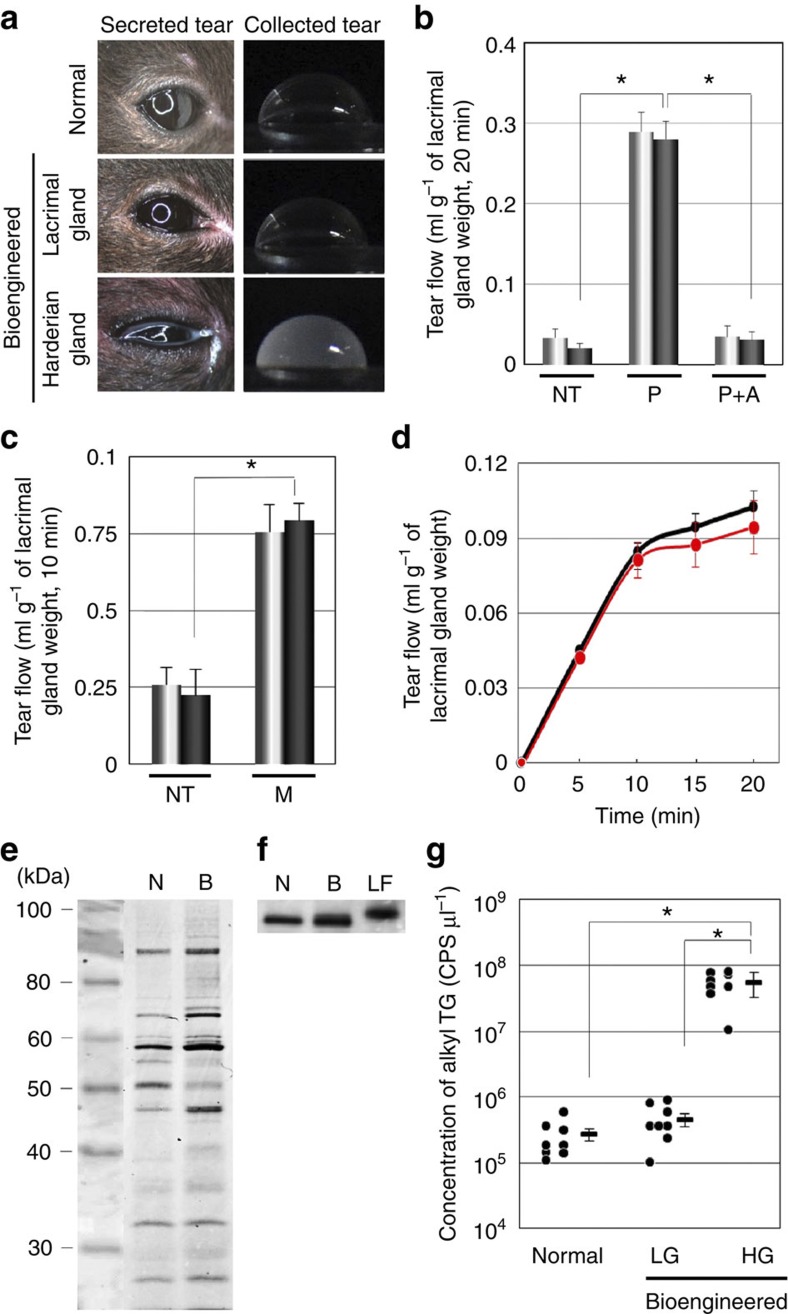
Functional analysis of the bioengineered gland. Functional analysis of the bioengineered gland. (**a**) Photographs of eyes after pilocarpine stimulation (left) and the collection of tear fluid (right), including a normal lacrimal gland (upper), a bioengineered lacrimal gland that was engrafted into a mouse (centre) and a bioengineered harderian gland that was engrafted into a mouse (lower). (**b**) Assessment of the tear flow in normal (light bars) and bioengineered (dark bars) lacrimal glands that were engrafted into mice at 30 days after surgery. Tear flow without pilocarpine stimulation (NT), with pilocarpine stimulation (P) and with pilocarpine and atropine stimulation (P+A) are shown. Error bars represent the s.e.m. (*n*=5). *P=0.0001 was regarded as statistically significant (Student’s *t*-test). NT, no-treatment; P, pilocarpine; and A, atropine. (**c**) Assessment of the tear flow and the response of normal (light bars) and bioengineered (dark bars) lacrimal glands that were engrafted into mice at 30 days after surgery. Tear flow without ocular surface stimulation (NT) and with ocular surface stimulation by menthol (M). Error bars represent the s.e.m. (*n*=6). *P=0.0005 was regarded as statistically significant (Student’s *t*-test). NT, no-treatment; M, ocular-surface stimulation by menthol. (**d**), The time course of tear flow in response to ocular-surface stimulation using menthol. The time course of tear flow for normal (black line) and bioengineered lacrimal glands (red line) after ocular-surface stimulation using menthol. Error bars represent the s.e.m. (*n*=6). (**e**) Tear proteins in secreted tear fluid were analysed using SDS-PAGE. N, normal; B, bioengineered. (**f**) Lactoferrin in tear fluid secreted from bioengineered lacrimal glands was detected using western blot analysis. N, normal; B, bioengineered; LF, recombinant lactoferrin. (**g**) The concentration of alkyl triglycerides in tear fluid secreted from normal mice, bioengineered lacrimal gland-engrafted mice and the bioengineered harderian gland-engrafted mice are shown. The data are presented as the median and the mean±s.e.m. (*n*=8). *P-values =0.0001 were regarded as statistically significant (Student’s *t*-test). CPS, count per second; LG, lacrimal gland; HG, harderian gland.

Appropriate nervous control of tear-fluid secretion is essential for the full function of bioengineered lacrimal glands and is required to protect the ocular surface. Therefore, we investigated whether bioengineered lacrimal glands would secrete proper tear fluid in response to nervous stimulation. After an intraperitoneal injection of pilocarpine, the ability to secrete tears (lacrimal flow) was determined. Observation of the ocular surface after pilocarpine stimulation revealed increased serous transparent tear secretion in the normal control and bioengineered lacrimal gland-engrafted mice, and increased amounts of turbid fluid were found in the bioengineered harderian gland-engrafted mice (Fig. 4a). The flow of tears after pilocarpine exposure was significantly increased in the mice with bioengineered lacrimal glands compared with lacrimal gland-defect mice, and there was no significant difference between the tear flow of mice with bioengineered lacrimal glands and that of the normal control mice. In addition, the anticholinergic agent atropine inhibited this effect (Fig. 4b). Lacrimation, which increases in response to mechanical, chemical and cooling stimulations to the ocular surface, has an important role in lacrimal gland function^30^,^31^,^32^,^33^. We next determined that bioengineered lacrimal glands could secret tears in response to cooling stimulation using menthol^33^. The tear flow from an engrafted bioengineered lacrimal gland after ocular-surface stimulation using menthol increased in line with that of the normal control mice (Fig. 4c,d). These findings indicated that bioengineered lacrimal glands received appropriate neural signals and had a secretory ability equivalent to that of the natural lacrimal glands. Next, we determined whether the tear fluid secreted from the bioengineered lacrimal glands contained the appropriate tear proteins, such as lactoferrin, which have an essential role in tear function. Tear-protein analysis revealed that the major bands, including lactoferrin, were detected in the tear fluid from bioengineered lacrimal glands (Fig. 4e,f). These results indicated that the bioengineered lacrimal gland had the ability to secrete tears containing tear proteins comparable to those of natural tears. We also analysed whether the tear fluid secreted from bioengineered harderian glands contained lipids such as alkyl triglycerides using reverse-phased liquid chromatography (LC) coupled with electrospray ionization (ESI)-quadrapole/time of flight hybrid mass spectrometry (QTOF-MS). The average±s.e.m of the concentrations of alkyl triglycerides in tear fluid secreted from control, bioengineered lacrimal and bioengineered harderian glands were 2.7±0.6 × 10^5^ counts per second (CPS) μl^−1^, 4.5±0.8 × 10^5^ CPS μl^−1^ and 5.5±1.0 × 10^7^ CPS μl^−1^, respectively (Fig. 4g). These findings indicated that bioengineered harderian glands could secrete the appropriate lipids. These results demonstrated that bioengineered lacrimal and harderian glands had functional secretory ability under appropriate neural control.

### Bioengineered lacrimal gland protects the ocular surface

**Figure 5:**
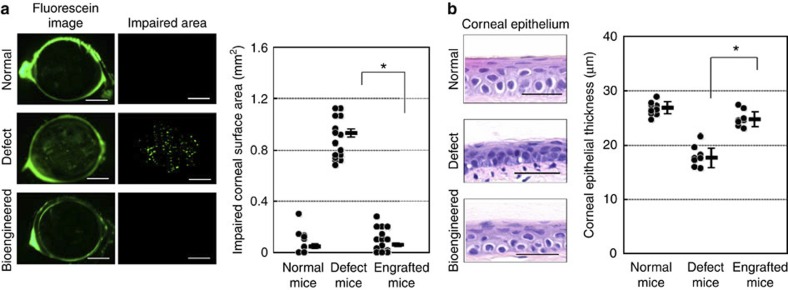
Ocular-surface protection function of the bioengineered lacrimal gland. Ocular-surface protection function of the bioengineered lacrimal gland. (**a**) Representative images (left) of the corneal surface of a normal lacrimal gland (upper), a lacrimal gland-defect mouse (centre) and a bioengineered lacrimal gland-engrafted mouse (lower). The punctate staining area in the 2.5-mm central corneal zone was measured using the Imaris software (right). Assessment of the impaired corneal surface area of a normal control (left), a lacrimal gland-defect mouse (centre) and a bioengineered lacrimal gland-engrafted mouse (right). The data are presented as the median and the mean±s.e.m. (*n*=20 for normal and the lacrimal gland-defect mice, 35 for the bioengineered lacrimal gland-engrafted mice). *P<0.0001 was regarded as statistically significant (Student’s *t*-test). Scale bar, 1 mm. (**b**) Representative microscopic images of the corneal epithelium at 60 days after surgery, including a same week-old control (upper), a lacrimal gland-defect mouse (centre) and a bioengineered lacrimal gland-engrafted mouse (lower). Scale bar, 25 μm. Assessment of the corneal epithelial thickness of the central cornea of the control (left), the lacrimal gland-defect mouse (centre) and the bioengineered lacrimal gland-engrafted mouse (right). The data are shown as the median and the mean±s.e.m. (*n*=11 for normal, 8 for the lacrimal gland-defect mice and the bioengineered lacrimal gland-engrafted mice). *P<0.0001 was regarded as statistically significant (Student’s *t*-test).

The goal of lacrimal gland-regenerative therapy is the restoration of an impaired ocular surface caused by DED. Tear loss from lacrimal gland dysfunction is a well-known cause of significant damage to the corneal epithelium. To determine the area of impaired corneal epithelium, fluorescein staining of the ocular surface was performed as previously described^34^. We investigated whether bioengineered lacrimal glands could protect the health of the ocular surface. The area of impaired corneal epithelium in bioengineered lacrimal gland-engrafted mice was significantly reduced compared with that of lacrimal gland-defect mice, and there was no significant difference between the areas of impaired corneal epithelium of bioengineered lacrimal gland-transplantation mice and those of normal control mice (Fig. 5a). Chronic tear loss has been shown to cause corneal epithelial thinning in dry-eye animal models^35^,^36^,^37^. Therefore, we next analysed whether the corneal thickness was maintained in the bioengineered lacrimal gland mice. The thickness of the corneal epithelium significantly decreased within 60 days after surgery in the lacrimal gland-defect mice, whereas the corneal thickness of bioengineered lacrimal gland-engrafted mice was equivalent to that of the normal control mice (Fig. 5b). These results indicate that our bioengineered lacrimal gland could successfully develop and achieve full lacrimal gland function and maintain a healthy ocular surface.

## Discussion

In this study, we successfully demonstrated that a bioengineered lacrimal gland replacement could restore the physiological functions of the lacrimal gland, including the production of a sufficient volume of tears and the protection of the ocular surface, via duct integration of an orthotopic engraftment of a bioengineered lacrimal gland germ into an adult extra-orbital lacrimal gland-defect model mouse, a model that mimics the ocular-surface damage of DED. These findings indicate that bioengineered lacrimal gland replacement via engraftment of a bioengineered lacrimal gland germ can restore lacrimal gland function.

The regeneration of lacrimal gland function is critical for developing a curative therapy for DED^17^. To develop tissue regeneration for salivary gland impairment using stem/progenitor cells^38^,^39^, tissue stem cells were identified from mice with salivary glands damaged by various phenomena, including radiation^25^, duct obstruction^23^ and cytokine injection^40^. Nestin- and Ki67-positive stem/progenitor cells capable of repairing interleukin-1-induced inflammation in murine-lacrimal glands^41^ and cells expressing stem cell markers such as c-kit, ABCG2 and ALDH1 in cultures of human lacrimal gland cells^42^ have been identified for tissue regeneration using stem cell transplantation. In contrast, we have proposed a novel approach for bioengineered organ replacement to restore organ function by the engraftment of a bioengineered organ germ *in vivo*^27^,^29^,^43^. In the present study, we further demonstrated that our organ-germ methods could be applied to not only teeth and hair follicles but also to secretory organs such as lacrimal and harderian glands. Bioengineered lacrimal and harderian gland germs, which were engrafted into extra-orbital lacrimal gland-defect model mice, developed *in vivo* into acini and duct with correct cell polarity including AQP5 expression. The bioengineered glands, the epithelial layers of which had extended along the guide to the recipient’s lacrimal excretory duct epithelium, secreted tears and lipids from the recipient’s excretory duct. Thus, these bioengineered lacrimal and harderian gland organ replacements have the potential to restore the function of impaired glands by ectopic or orthotopic engraftment of their bioengineered germs.

In the lacrimal and harderian glands, the acini and ductal system, which is the functional unit of secretory organs, such as the lacrimal glands, salivary glands and pancreas, has an important role in physiological secretion^2^ through interaction with the surrounding tissues, including connective myoepithelial cells and nerve fibres. The 3D histo-architecture of these glands is achieved by the differentiation of various cell types and by the branching morphogenesis that occurs during organogenesis^44^,^45^,^46^. In organ regeneration by orthotopic transplantation of a bioengineered organ germ, which can reproduce the developmental process during embryogenesis, the bioengineered tooth or hair-follicle organ can establish interactions between connective tissues, such as the periodontal ligament and alveolar bone in the tooth or the arrector pili muscles in the hair follicle, and nerve fibres^28^,^29^,^47^. Lacrimation in response to noxious stimulation of the ocular surface is important for ocular-surface protection and the functioning of the lacrimal gland^30^,^31^. Cooling stimulation of the ocular surface induced lacrimation via a neural pathway initiated by the activation of corneal cool cells^33^. Our current study demonstrated that bioengineered lacrimal and harderian gland germs, which reproduce their developmental process, could develop by engraftment with integrated myoepithelial cells and nerves *in vivo* and then secrete tears in response to ocular surface stimulation using menthol. Our findings indicated that the bioengineered lacrimal gland could reconstruct a 3D secretory system that was integrated into the recipient’s tissues.

The tear film, which is composed of lipid, aqueous and mucin layers, has many physiological functions and serves as a mechanical and antimicrobial barrier to protect the ocular surface and to ensure an optically refractive surface^3^. The aqueous component contains electrolytes, water and various proteins, including peptides and glycoproteins, and is secreted primarily by the lacrimal glands. The tear proteins, including lactoferrin, the main tear protein produced by the lacrimal gland, have an essential role in tear function, which includes tear stability, moisturization, wound healing and antibiotic effects^2^,^3^,^4^. To restore tear function, albumin^48^ and autologous serum^49^ have been pursued as tear substitutes for severe ocular-surface disorders. In our study, we demonstrated that the tear fluid secreted from bioengineered lacrimal glands contained major tear proteins, including lactoferrin, that are almost identical to those found in natural tears. These bioengineered glands also protected the ocular surface from damage in the lacrimal gland-defect mouse model. Tear lipids are another critical component necessary for tear function, and these lipids stabilize tears and prevent evaporation^15^. Dysfunction of the lipid-secretory organs, such as the meibomian gland in humans and the harderian gland in mice^6^, causes DED^50^, and a supplemental lipid treatment has been reported to improve meibomian gland dysfunction^51^. Our findings suggested that a bioengineered harderian gland could supply a significant amount of alkyl TG in the tear lipids by ectopic engraftment in our experimental model. The analysis of those components such as detail minor proteins in the tears from bioengineered glands would provide useful information, including tear stability, antimicrobial effect and barrier function, for the restoration of physiological tear function by organ regeneration.

In conclusion, the current study provides novel evidence for the successful replacement of a fully functional lacrimal gland via engraftment of a bioengineered germ to restore full lacrimal gland function. Further studies on the identification of stem cells, including adult tissue stem cells, embryonic stem cells and inductive pluripotent stem cells, as cell sources for bioengineered lacrimal and harderian gland germs will contribute to the development of lacrimal gland organ regeneration. It is also essential to study the potential clinical application of these therapies, including the successful orthotopic or ectopic engraftment of the bioengineered organ germ into diseased conditions such as inflammation, Sjogren’s syndrome, ocular cicatricial pemphigoid and aging in humans in the future.

## Methods

### Animals

C57BL/6 mice were purchased from Japan SLC Inc. (Shizuoka, Japan). CL57BL/6-TgN (act-EGFP) OsbC14-Y01-FM131 and B6.Cg-Tg (CAG-DsRed*MST) 1Nagy/J mice were obtained from SLC Inc. and the RIKEN Bioresource Centre (Tsukuba, Japan), respectively. R26-H2B-EGFP KI mice were kindly provided by Professor Fujimori, National Institute for Basic Biology (Aichi, Japan). The care and handling of the animals were performed in accordance with the NIH guidelines. All of the experimental protocols were approved by the Animal Care and Use Committee of the Tokyo University of Science.

### Reconstitution of bioengineered gland germs with single cells

Lacrimal gland germs and harderian gland germs isolated from embryonic day-16.5 male and -female mice were treated with 50 U ml^−1^ dispase (BD, Franklin Lakes, NJ, USA) for 1.5 min at room temperature. Epithelial and mesenchymal tissues were separated. The epithelial tissues were treated twice with 100 U ml^−1^ collagenase I (Worthington, Lakewood, NJ, USA) in Ca^2+^- and Mg^2+^-phosphate-buffered saline (PBS(−)) at 37 °C for 10 min, treated with 0.25% trypsin (Sigma, St. Louis, MO, USA) in PBS(−) for 5 min at 37 °C and then dissociated into single epithelial cells by gentle pipetting. Mesenchymal single cells were prepared by treatment with PBS(−) supplemented with 0.25% trypsin and 50 U ml^−1^ collagenase I at 37 °C for 10 min. Single epithelial and mesenchymal cells were precipitated by centrifugation, and the supernatants were removed. The bioengineered gland germs were reconstituted using our previously described 3D cell-manipulation method^43^. Briefly, mesenchymal cells were injected into collagen drop using micropipette. Subsequently, epithelial cells were injected into the same collagen drop adjacent to the mesenchymal cell aggregate. To induce interepithelial tissue connections between the host lacrimal excretory duct and the bioengineered germs, a PGA monofilament thread guide (9-0 PGA absorbable surgical suture: Gunze, Kyoto, Japan) was appended to a bioengineered germ by inserting the guide through the epithelial and mesenchymal portions. Bioengineered germs were placed on a cell-culture insert (0.4 μm pore diameter, BD) and incubated at 37 °C for 3 days in DMEM/F-12 (1:1 mixture of Dulbecco’s Modified Eagle’s Medium and Ham’s F-12; Kohjin Bio, Saitama, Japan) supplemented with 10% foetal bovine serum (GIBCO, Grand Island, NY, USA), 100 U ml^−1^ penicillin (Sigma) and 100 μg ml^−1^ streptomycin.

### Engraftment procedure of bioengineered transplant

To prepare the lacrimal gland-defect model mice, the extra-orbital lacrimal gland of 7-week-old C57BL/6 female mice was extracted under deep anaesthesia. Then, the bioengineered gland germ was engrafted into the lacrimal gland-defect model mouse. A PGA monofilament was inserted into the host lacrimal excretory duct, and collagen gel containing the bioengineered germ was sutured to the massetermuscle with 8-0 nylon thread (8-0 black nylon 4 mm 1/2R, Bear Medic Corp., Ibaraki, Japan).

### Dye injection for the analysis of the duct connection

Thirty, or fourteen days after surgery, 6 mg ml^−1^ Evans blue dye (Sigma) or FITC (Dojindo, Kumamoto, Japan)-conjugated gelatin was injected into the host lacrimal duct using a FemtoJet microinjector (Eppendorf, Hamburg, Germany) under a SteREO Lumar V12 microscope (Carl Zeiss, Oberkochen, Germany).

### Immunohistochemical analysis

For fluorescence immunohistochemistry, the tissues were fixed in Mildform 10 N (Wako, Osaka, Japan) overnight at 4 °C, and frozen sections (10 and 100 μm) were prepared and immunostained. The primary antibodies were as follows: E-cadherin (1:50, mouse, BD); AQP5 (1:100, rabbit, Millipore, Billerica, MA, USA); calponin (1:250, rabbit, Abcam, Cambridge, MA, USA); and neurofilament-H (1:500, rat, Millipore) diluted in blocking solution (PBS(−) containing 1% bovine serum albumin and 0.01% TX-100). The primary antibodies were detected using highly cross-absorbed Alexa Fluor 594 Goat Anti-Rabbit IgG (H+L) (1:500, Life Technologies, Carlsbad, CA, USA) and Alexa Fluor 488 Goat Anti-Rat IgG (H+L) (1:500, Life Technologies) for 1 h at room temperature together with Hoechst 33342 dye (1:500, Life Technologies). Fluorescence microscopy images were obtained using a laser confocal microscope (LSM780; Carl Zeiss). To detect lactoferrin, 5-μm-thick paraffin sections were treated with rabbit polyclonal antiserum directed against lactoferrin (1:200, Millipore) as the primary antibody and a biotinylated goat anti-rabbit secondary antibody (Histofine Kit; Nichirei Bio, Tokyo, Japan). Immunoreactivity was detected using streptavidin–peroxidase (Nichirei Bio) and 3,3′-diaminobenzidine (Millipore). The sections were counterstained using hematoxylin and observed using an Axioimager A1 microscope (Carl Zeiss).

### Oil-red O staining

Frozen sections were washed with propylene glycol for 3 min and then soaked in Oil-Red O (Sigma) staining solution for 30 min. The sections were counterstained with hematoxylin and observed using an Axioimager A1 microscope (Carl Zeiss).

### Collection and measurement of tear-fluid secretion volume

Tears were collected from the eyelid margin without touching the eye using a 0.5-μl micropipette (Drummond Scientific, PA, USA) 20 min after stimulation by intraperitoneal injection of 300 μg of pilocarpine kg^−1^ body weight under anaesthesia, or after ocular surface stimulation by 0.1 μM menthol (Sigma) without anaesthesia. The mice were placed in a modified DecapiCone restraint (MDC-200; Braintree Scientific, Braintree, MA) with sufficient acclimation. Following baseline measurements, 10 μl of 0.1 mM menthol were applied directly to the ocular surface with a micropipette. After 2 min, the fluid was wicked away with a Kimwipe (Fisher Scientific, Pittsburgh, PA) by lightly touching the tear meniscus at the lateral canthus. The tear was collected 15 min after removal of the fluid^33^.

### Tear-protein analysis

A 0.25-μl aliquot of tear fluid from natural or bioengineered glands and a 0.4-ng sample of recombinant lactoferrin (kindly provided by Dr Morishita, Lion Corp., Tokyo, Japan) were subjected to 10% SDS-PAGE. The proteins were visualized using SYPRO orange protein gel stain (Life Technologies) for 30 min. The proteins were transferred on polyvinylidene difluoride membranes using a semidry transblot system (Bio-Rad, Philadelphia, PA, USA). Western blotting was performed by standard procedures using an antibody against mouse lactoferrin (1:10,000, Millipore) and peroxidase-conjugated donkey anti-rabbit IgG (1:10,000, Jackson ImmunoResearch Laboratories, West Grove, PA, USA) diluted in Can Get Signal Immunoreaction Enhancer Solution (Toyobo, Osaka, Japan). The immunoreactive proteins were detected using the ECL plus Western Blotting Detection Kit (Roche, Basel, Switzerland). Images were captured using a Luminescent Image Analyzer LAS-3000 (Fujifilm, Tokyo, Japan) and processed with Multi Gauge software (Fujifilm).

### Tear-lipid analysis

Total lipids were extracted from 1 μl of tear with 100 μl of methanol containing internal standards for 2.5 h at room temperature and centrifuged (2,000 × *g*) for 5 min at room temperature^52^. The collected supernatants were dried under a gentle stream of nitrogen and re-dissolved with 100 μl of acetonitrile/methanol/water (1:1:3; v/v/v). Reversed-phase (LC) separation was performed with an ACQUITY UPLC HSS T3 column (50 × 2.1 mm i.d., Waters Corporation, Milford, MA, USA) at 45 °C. The mobile phase was prepared by mixing the solvents acetonitrile/methanol/water (1:1:3; v/v/v) (5 mM ammonium formate) and isopropanol (5 mM ammonium formate). The mobile phase was pumped at a flow rate of 300 μl min^−1^. LC/ESI-MS analysis was performed using a TripleTOF 5600, QTOF-MS (AB SCIEX, Foster City, CA, USA) with an Agilent 1290 Infinity LC system (Agilent Technologies, Loveland, CO, USA) in the positive ion mode^53^. The parameter settings were as follows: 5.5 kV for the ion-spray voltage floating, 500 °C for the ion-source temperature, 10 V for the collision energy and *m/z* 100–1,250 for the scan range.

### Calculation of impaired corneal epithelial area

Fluorescein staining of the corneal epithelium was used as a diagnostic tool to study the effect of desiccating stress on the ocular surface of the mice. Using a micropipette, 0.7 μl of 2.5% fluorescein (Sigma) was applied to the inferior conjunctival sac of the eye^54^. The punctuate staining pattern on the corneal surface was photographed using a SteREO Lumar V12 microscope (Carl Zeiss). The area of punctate staining within a radius of 2.5 mm in the central corneal zone of each eye was measured using Imaris software (Bitplane, Zurich, Switzerland).

### Histochemical analysis of corneal epithelial thickness

The eyeballs were dissected, fixed in Mildform 10 N (Wako) and processed for standard paraffin embedding, and 5-μm sagittal sections were obtained. The tissue sections were stained with hematoxylin–eosin and observed using an Axioimager A1 microscope (Carl Zeiss). The thickness of the corneal epithelium of the central cornea was measured using the AxioVision software (Carl Zeiss).

### Statistical analysis

We presented data as the mean±s.e.m. We used two-tailed Student's *t*-tests to determine *P*-values for statistical significance.

## Author contributions

T.T., K.T., T.K. and M.H. designed the research plan; M.H., M.O., Y.S., K.Y. and K.I. performed the experiments; M.H., S.S. and M. Oshima developed new assay methods and discussed the results; M.H., Y.S., K.I., K.Y. and M.O. analysed the data; and M.H. and T.T. wrote the paper.

## Additional information

**How to cite this article:** Hirayama, M. *et al*. Functional lacrimal gland regeneration by transplantation of a bioengineered organ germ. *Nat. Commun.* 4:2497 doi: 10.1038/ncomms3497(2013).
